# MicroRNA-29a represses osteoclast formation and protects against osteoporosis by regulating PCAF-mediated RANKL and CXCL12

**DOI:** 10.1038/s41419-019-1942-1

**Published:** 2019-09-23

**Authors:** Wei-Shiung Lian, Jih-Yang Ko, Yu-Shan Chen, Huei-Jing Ke, Chin-Kuei Hsieh, Chung-Wen Kuo, Shao-Yu Wang, Bo-Wun Huang, Jung-Ge Tseng, Feng-Sheng Wang

**Affiliations:** 1 Core Laboratory for Phenomics and Diagnostic, Kaohsiung Chang Gung Memorial Hospital, Kaohsiung, Taiwan; 2 Department of Medical Research, Kaohsiung Chang Gung Memorial Hospital, Kaohsiung, Taiwan; 3 Department of Orthopedic Surgery, Kaohsiung Chang Gung Memorial Hospital, Kaohsiung, Taiwan; 4 Department of Mechanical Engineering, Cheng Shiu University, Kaohsiung, Taiwan; 5 Department of Leisure and Sports Management, Cheng Shiu University, Kaohsiung, Taiwan; 6 Graduate Institute of Clinical Medical Sciences, Chang Gung University College of Medicine, Kaohsiung, Taiwan

**Keywords:** Mechanisms of disease, Osteoporosis

## Abstract

Osteoporosis deteriorates bone mass and biomechanical strength, becoming a life-threatening cause to the elderly. MicroRNA is known to regulate tissue remodeling; however, its role in the development of osteoporosis remains elusive. In this study, we uncovered that silencing miR-29a expression decreased mineralized matrix production in osteogenic cells, whereas osteoclast differentiation and pit formation were upregulated in bone marrow macrophages as co-incubated with the osteogenic cells in transwell plates. In vivo, decreased miR-29a expression occurred in ovariectomy-mediated osteoporotic skeletons. Mice overexpressing miR-29a in osteoblasts driven by osteocalcin promoter (miR-29aTg/OCN) displayed higher bone mineral density, trabecular volume and mineral acquisition than wild-type mice. The estrogen deficiency-induced loss of bone mass, trabecular morphometry, mechanical properties, mineral accretion and osteogenesis of bone marrow mesenchymal cells were compromised in miR-29aTg/OCN mice. miR-29a overexpression also attenuated the estrogen loss-mediated excessive osteoclast surface histopathology, osteoclast formation of bone marrow macrophages, receptor activator nuclear factor-κ ligand (RANKL) and C–X–C motif chemokine ligand 12 (CXCL12) expression. Treatment with miR-29a precursor improved the ovariectomy-mediated skeletal deterioration and biomechanical property loss. Mechanistically, miR-29a inhibited RANKL secretion in osteoblasts through binding to 3′-UTR of RANKL. It also suppressed the histone acetyltransferase PCAF-mediated acetylation of lysine 27 in histone 3 (H3K27ac) and decreased the H3K27ac enrichment in CXCL12 promoters. Taken together, miR-29a signaling in osteogenic cells protects bone tissue from osteoporosis through repressing osteoclast regulators RANKL and CXCL12 to reduce osteoclastogenic differentiation. Arrays of analyses shed new light on the miR-29a regulation of crosstalk between osteogenic and osteoclastogenic cells. We also highlight that increasing miR-29a function in osteoblasts is beneficial for bone anabolism to fend off estrogen deficiency-induced excessive osteoclastic resorption and osteoporosis.

## Introduction

Bone mass homeostasis is sophisticatedly integrated by dynamic processes of bone formation and resorption^1^. Upon deleterious extracellular stresses, excessive bone erosion causes an extremely meager bone mineral density along with a fragile microarchitecture, accelerating the development of osteoporosis. This chronic bone disease overwhelms aged patients’ activity, independence and even survival^2,3^. With respect to the bone cell function to skeletal metabolism, osteogenic cells are essential to gain mineralized matrices, on the contrary, osteoclasts are responsible for remodeling bone microstructure^4^. The former also produces a plethora of cytokines and chemokines, like receptor activator nuclear factor-κ ligand (RANKL), osteoprotegrin (OPG), and C–X–C motif chemokine ligand 12 (CXCL12), regulating osteoclast formation and resorption capacity^5–7^. The molecular events underlying the excessive resorption in osteoporotic bone have been not well elucidated.

MicroRNA act as epigenetic regulators to interfere with mRNA expression through binding the 3′-untranslated region (3′-UTR) of the target, and thus change biological activity^8^. Increasing evidence reveals arrays of microRNA actions to osteoblast function and osteoclast behavior during bone development, degeneration and metastasis. For example, serum miR-22-3p, miR-328-3p and let-7g-5p levels are correlated with osteoporotic fracture in women^9^. Serum miR-550a-5p, miR-188-3p and miR-382-3p are discriminative signatures for skeletal fracture in menopausal females with diabetes^10^. In experimental osteoporosis models, mice deficient in miR-188 show minor responses to the age-mediated marrow fat overproduction and bone loss^11^. Silencing miR-503 ramps up osteoclast formation of CD14-positive peripheral blood mononuclear cells in human, aggravating the development of osteoporosis in ovariectomized mice^12^. Osteoclast-specific miR-34a knockout mice display severe bone loss along with increased bone resorption, whereas estrogen deficiency-mediated osteoporosis and skin cancer-mediated bone metastasis are compromised in mice overexpressing miR-34a^13^.

The miR-29 family participates in mineralized matrix production and excessive calcified tissue development. Decreased serum miR-29 levels are correlated with vertebral fracture in postmenopausal women^14^, whereas increased miR-29 expression is associated with high osteogenic differentiation potential of mesenchymal stem cells from human osteoarthritic subchondral bone^15^, and the development of secondary hyperthyroidism-mediated bone disorder in rats with chronic kidney disease^16^. Knocking down miR-29a reduces calcified matrix production in vascular smooth muscle cells^17^. We previously revealed that miR-29a signaling protected skeletal tissue from glucocorticoid excess-induced fatty marrow^18^. Its biological roles in the development of estrogen deficiency-mediated bone loss remains elusive.

This study aims to utilize osteoblast-specific miR-29a transgenic mice to investigate the miR-29a action to osteoclast formation, verify whether it changes estrogen deficiency-induced bone loss, and elucidate the mechanistic underlying osteoblastic miR-29a regulation of osteoclastogenesis.

## Results

### miR-29a regulated osteoblast–osteoclast interaction

**Fig. 1:**
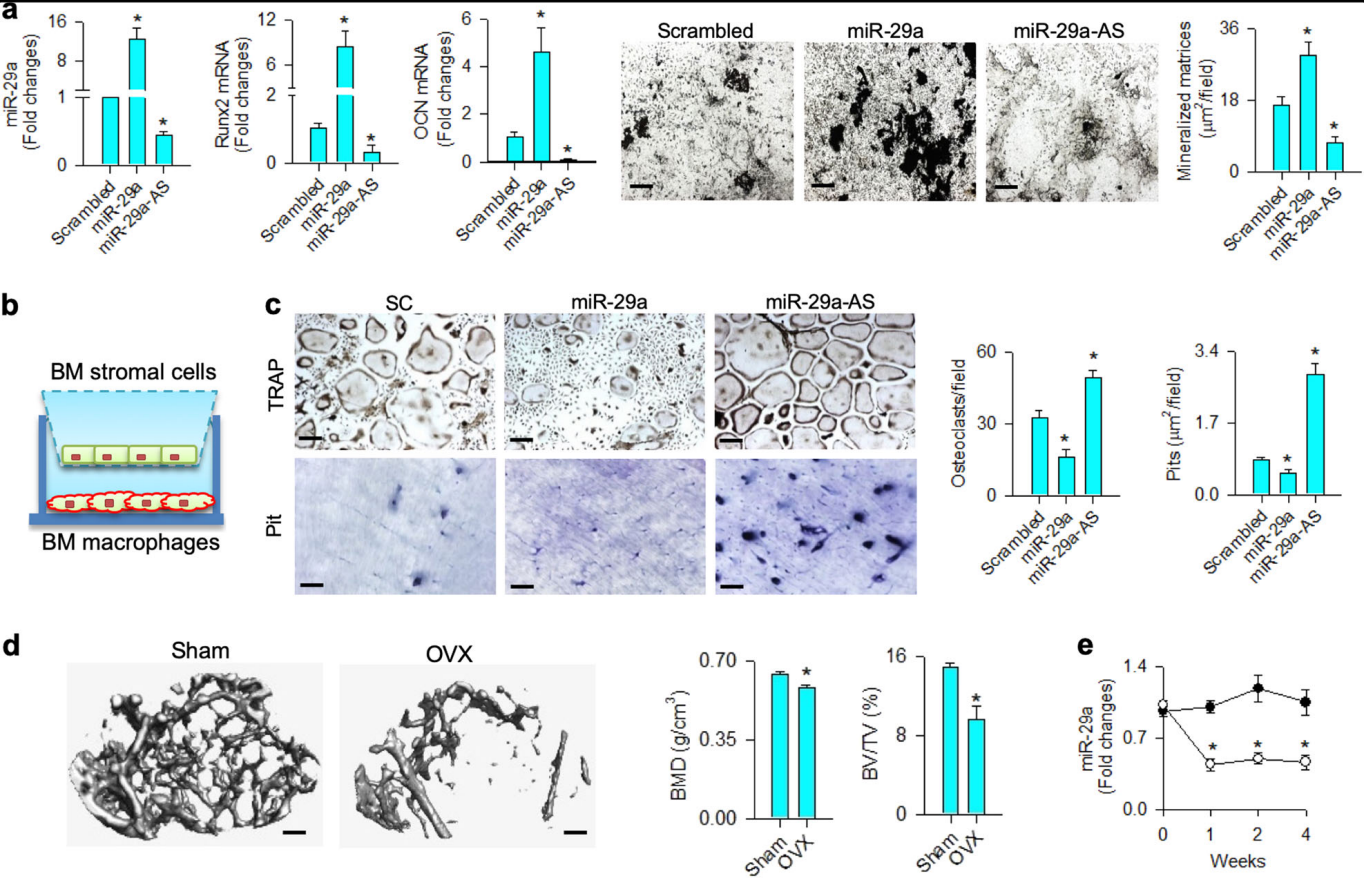
Analysis of miR-29a actions to osteogenic and osteoclastogenic differentiation. Increased Runx2 and osteocalcin expression and von Kossa-stained mineralized matrix formation in miR-29a-transfected bone marrow mesenchymal cells (**a**); scale bar: 40 μm. Schematic drawing of bone marrow macrophages and mesenchymal cells co-incubated in transwell plates (**b**). Increasing miR-29a in osteoblasts decreased TRAP-stained osteoclast differentiation (scale bar: 8 μm) and pit formation (scale bar: 20 μm) of bone marrow macrophages (**c**). The data of in vitro model are expressed as mean ± SEM calculated from four experiments. Asterisks * indicate significant difference from the scrambled control. Sparse trabecular microstructure (scale bar: 5 mm) along with decreased BMD, BV/TV (**d**), and miR-29a expression in bone tissue (**e**) occurred in ovariectomized mice. Investigations are expressed mean ± SEM calculated from six mice. Asterisks * indicate significant difference from sham controls

First, we characterized which role miR-29a may play in osteogenic differentiation. Bone marrow mesenchymal stem cells were transfected with miR-29a precursor or antisense oligonucleotide (miR-29a-AS). Forced miR-29a expression increased the osteogenic marker Runx2 and osteocalcin expression along with upregulated mineralized matrix production as evident from von Kossa staining, whereas knockdown of miR-29a reduced osteogenic activity (Fig. 1a).

Transwell culture systems were adopted to examine whether the miR-29a-modulated osteogenic cells changed osteoclast formation. Bone marrow mesenchymal cells were incubated in the upper wells of transwell plates, bone marrow macrophages were seeded in the lower wells containing M-CSF and RANKL (Fig. 1b). miR-29a-transfected osteogenic cells resulted in a significant decrease in osteoclast differentiation as evident from tartrate-resistant acid phosphatase (TRAP) staining. They also significantly downregulated resorption capacity of osteoclast as fewer pits were formed on bone cortical slices than the scrambled controls. On the contrary, miR-29a-AS-treated osteogenic cells promoted osteoclastogenic differentiation capacity and pit formation of macrophages (Fig. 1c).

Next to cell culture models, ovariectomized mice were utilized to mimic estrogen deficiency-mediated bone loss and test whether miR-29a expression was changed in bone tissue. μCT images revealed sparse trabecular microstructure along with significant decreases in bone mineral density (BMD) and trabecular bone volume (BV/TV) at 4 weeks postoperatively (Fig. 1d). Estrogen deficiency also caused significant reductions in miR-29a expression in bone tissue throughout the study period as compared with the sham controls (Fig. 1e).

### Osteoblast-specific miR-29a transgenic mice showed high bone mass

**Fig. 2:**
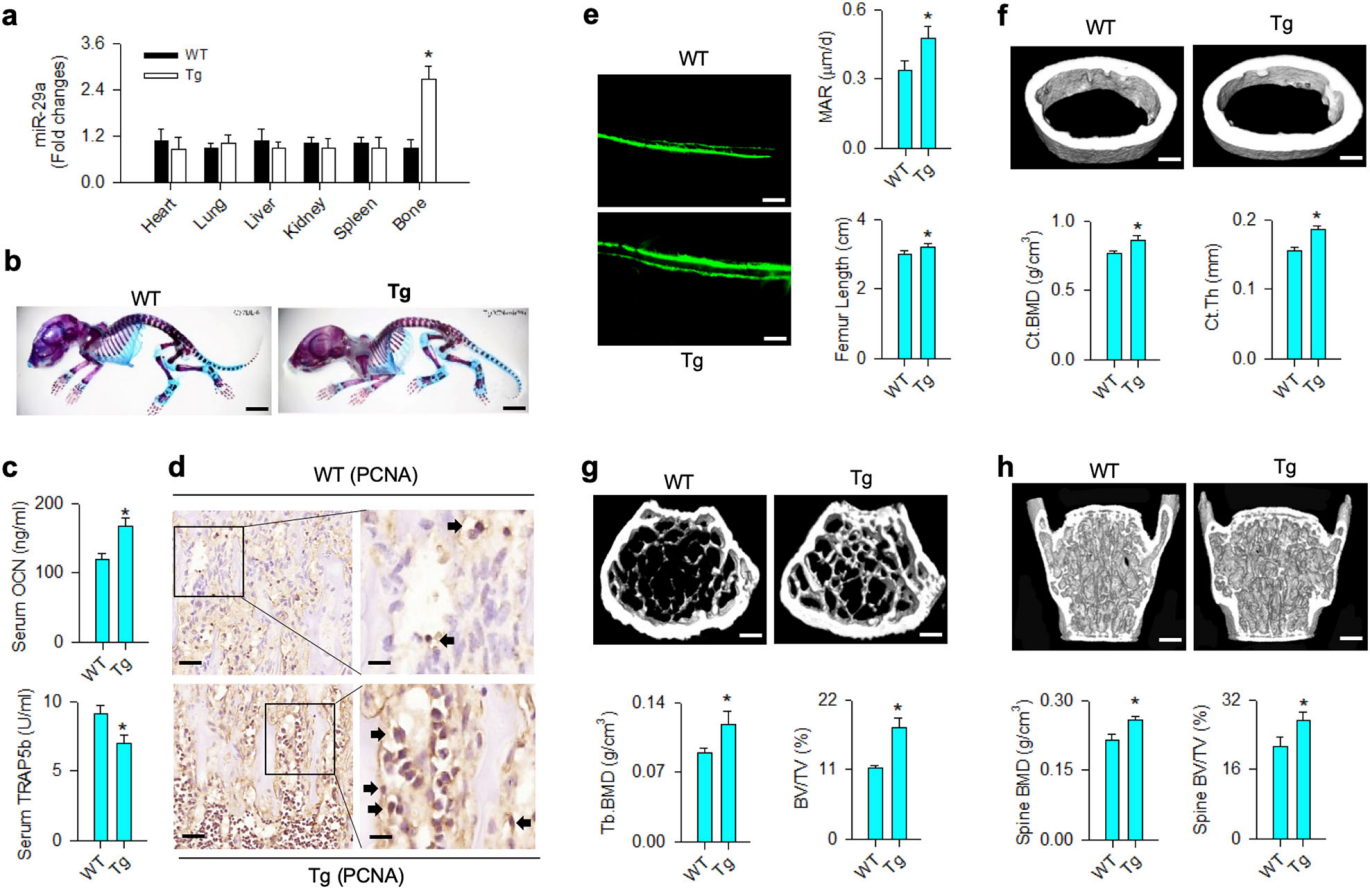
Skeletal phenotypes and bone microstructure of miR-29aTg/OCN mice. Increased miR-29a expression solely occurred in bone tissue in miR-29aTg/OCN mice (**a**). Macroscopic morphology of 7-day-old miR-29aTg/OCN mice was similar to wild-type mice (**b**); scale bar: 20 mm. Increased serum osteocalcin and reduced TRAP5b levels (**c**) and strong PCNA immunostaining (**d**) in osteoblasts in miR-29aTg/OCN mice; scale bar: 30 μm (low magnification); 10 μm (high magnification). Increased fluorescent calcein accumulation and MAR along with femur length in 8-week-old miR-29aTg/OCN mice (**e**); scale bar: 30 μm. Significant increases in cortical BMD and Ct.Th (**f**), trabecular BMD and BV/TV (**g**) together with spinal BMD and BV/TV (**h**) in miR-29aTg/OCN mice; scale bar: 5 mm. The data are expressed mean ± SEM calculated from six mice. Asterisks * indicate significant difference from wild-type mice

The analysis of in vitro models and experimental osteoporosis prompted us to breed transgenic mice overexpressing miR-29a in osteoblasts driven by osteocalcin promoter (miR-29aTg/OCN). RT-quantitative PCR confirmed that miR-29a expression in skeleton rather than other tissues was significantly increased in miR-29aTg/OCN mice as compared with WT mice (Fig. 2a). In 7-day-old miR-29aTg/OCN mice, macroscopic morphology, like the skull, chest cage spine and long bone, was similar to WT mice as evident from whole-mount staining (Fig. 2b). At 8 weeks old, significantly increased serum osteocalcin levels and reduced serum TRAP5b abundances (Fig. 2c) along with intensive osteoblast growth histology as evident from proliferating cell nuclear antigen (PCNA) immunostaining (Fig. 2d) occurred in miR-29aTg/OCN mice. Fluorescent calcein accumulation, mineral acquisition rate and femur length of miR-29aTg/OCN mice were also significantly higher than WT mice (Fig. 2e). In addition, miR-29a overexpression significantly augmented cortical BMD (Fig. 2f) and trabecular BMD of femurs (Fig. 2g) and L1–L5 vertebrae (Fig. 2h) along with increased BV/TV and cortical thickness (Ct.Th) as evident from μCT analyses.

### miR-29a attenuated the ovariectomy-induced bone mass and biomechanics loss

**Fig. 3:**
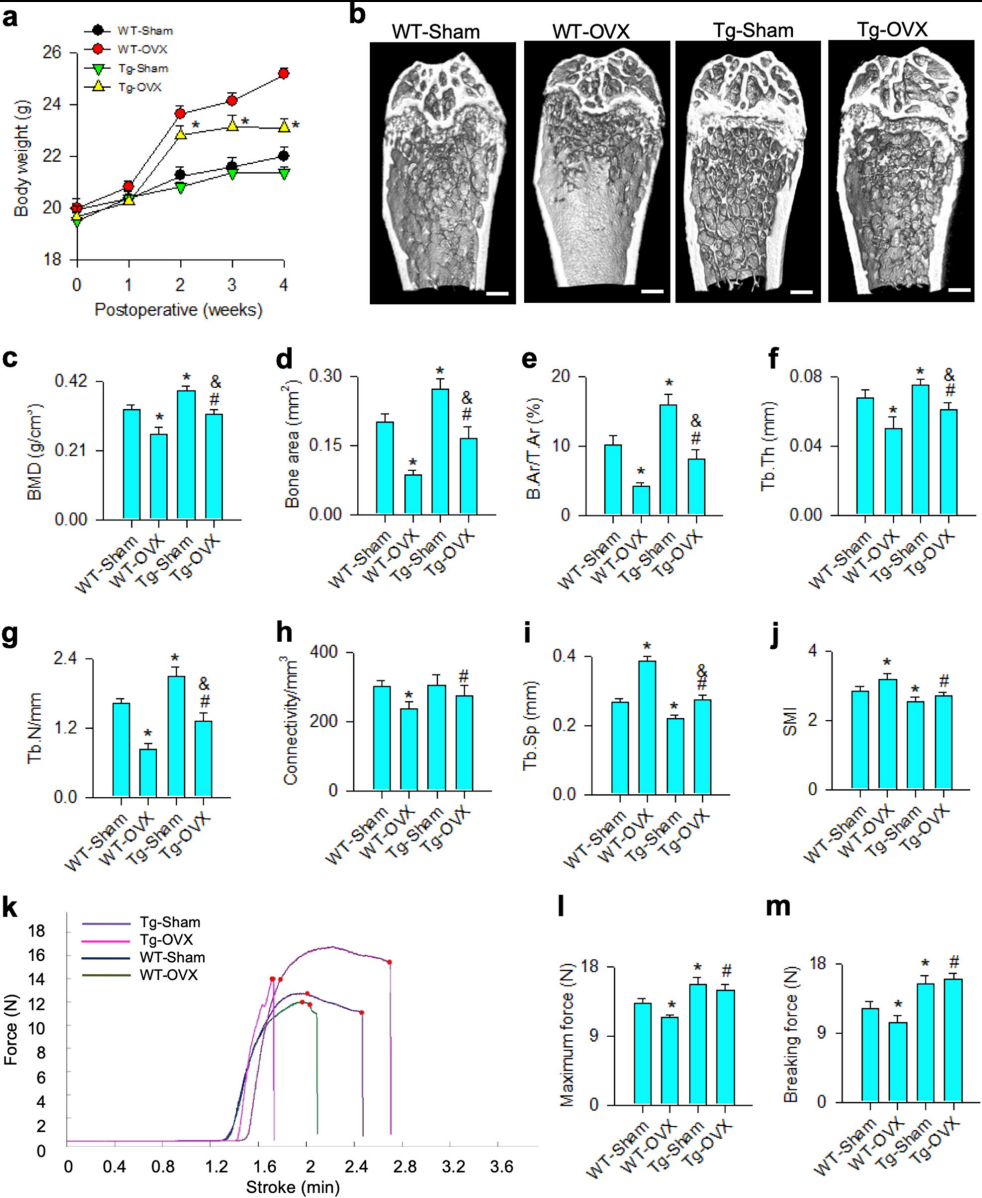
Analysis of bone mass and biomechanical property of skeletal tissue. miR-29a overexpression reduced the ovariectomy-induced excessive body weight gain (**a**) and retained abundant trabecular microstructure (**b**); scale bar: 5 mm. The ovariectomy-induced loss of BMD (**c**), bone area (**d**), B.Ar/T.Ar (**e**), Tb.Th (**f**), Tb.N (**g**), and connectivity (**h**) were attenuated in miR-29aTg/OCN mice, whereas Tb.Sp (**i**) and SMI (**j**) were improved. Load-displacement curve (**k**) showed minor responses to ovariectomy-induced downregulation of maximum force (**l**) and breaking force (**m**) in miR-29aTg/OCN mice. The data are expressed mean ± SEM calculated from six mice. Asterisks * and hashtags # indicate significant difference from sham controls and OVX, respectively. Ampersands & indicate significant difference from Tg-Sham and Tg-OVX

Given that miR-29a overexpression increased bone formation, we further tested whether it changed estrogen deficiency-induced osteoporosis. Body weight of ovariectomized miR-29aTg/OCN mice was less than ovariectomized WT mice by 2 weeks postoperatively (Fig. 3a). Bone tissue in ovariectomized WT mice showed sparse trabecular microstructure, whereas abundant trabecular bone existed in ovariectomized miR-29aTg/OCN mice (Fig. 3b). miR-29a overexpression significantly compromised the estrogen deficiency-mediated loss of BMD (Fig. 3c) and morphometric characteristics of trabecular bone, like bone area (Fig. 3d), B.Ar/T.Ar (Fig. 3e), Tb.Th (Fig. 3f), Tb.N (Fig. 3g) and connectivity (Fig. 3h). It also reduced the ovariectomy aggravation of Tb.Sp (Fig. 3i) and SMI (Fig. 3j).

We performed material tests to characterize mechanical properties of bone tissue (Fig. 3k). Maximum force (Fig. 3l) and breaking force (Fig. 3m) of femurs were significantly reduced in ovariectomized WT mice. miR-29a overexpression significantly increased the baseline mechanical properties and compromised the estrogen deficiency-mediated loss of biomechanical profiles.

### miR-29a increased bone formation and attenuated osteoclastic resorption

**Fig. 4:**
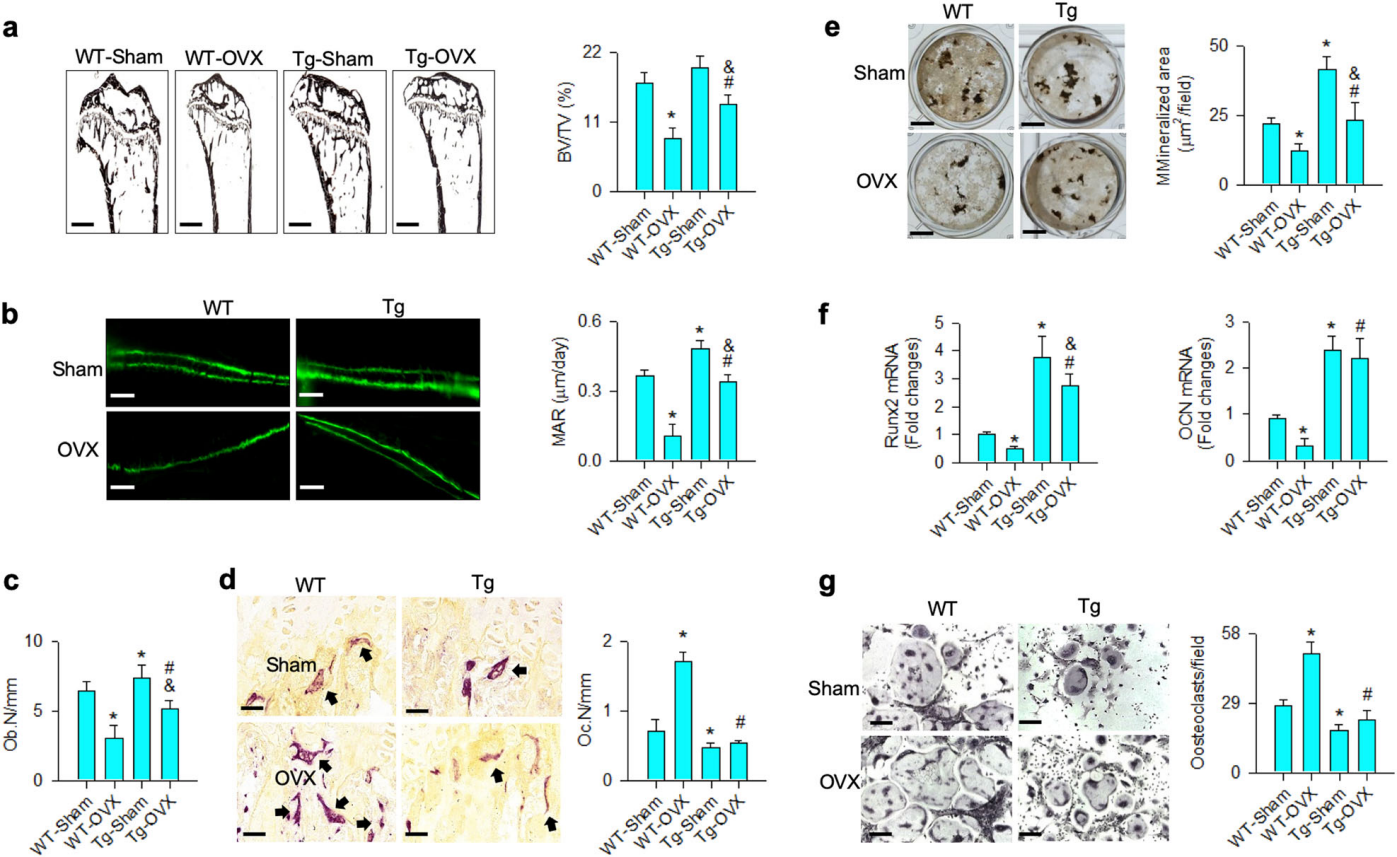
Analysis of bone histology and ex vivo osteogenesis and osteoclastogenesis. miR-29a overexpression repressed the ovariectomy-mediated loss of trabecular histology (scale bar: 120 μm), BV/TV (**a**), fluorescent calcein labeling (scale bar: 30 μm), MAR (**b**) and Ob.N (**c**), as well as compromised excessive TRAP-positive osteoclast distribution (scale bar: 8 μm) and Oc.N (**d**). miR-29aTg/OCN mice showed minor response to the ovariectomy-induced loss of mineralized matrix formation (scale bar: 7 mm) (**e**), Runx2 and osteocalcin expression (**f**) of primary bone marrow mesenchymal cells and downregulated osteoclast formation of primary bone marrow macrophages (**g**); scale bar: 8 μm. The data are expressed mean ± SEM calculated from six mice. Asterisks * and hashtags # indicate significant difference from sham controls and OVX, respectively. Ampersands & indicate significant difference from Tg-Sham and Tg-OVX

In addition, bone tissue in ovariectomized WT mice showed histopathological features of severe trabecular loss (Fig. 4a) along with reduced bone mineral accretion (Fig. 4b) and decreased osteoblast number (Ob.N; Fig. 4c), but exhibited increased osteoclast number (Oc.N; Fig. 4d) as compared with sham controls. The estrogen deficiency-induced loss of trabecular histology and high osteoclast distribution were attenuated in ovariectomized miR-29aTg/OCN mice.

The improved bone formation and resorption in ovariectomized miR-29aTg/OCN mice reasoned us to test osteogenic and osteoclastogenic differentiation capacity in osteoporotic bone. Primary mesenchymal cells and macrophages from bone marrow were isolated and incubated in osteogenic and osteoclastogenic media, respectively. Consistent with the histological analyses, miR-29a overexpression significantly attenuated the estrogen deficiency-mediated loss of mineralized matrix accumulation (Fig. 4e) and decreased osteogenic markers Runx2 and osteocalcin expression (Fig. 4f) of bone marrow mesenchymal cells. It also significantly repressed osteoclast formation of bone marrow macrophages from ovariectomized mice (Fig. 4g).

### miR-29a precursor ameliorated osteoporosis development

**Fig. 5:**
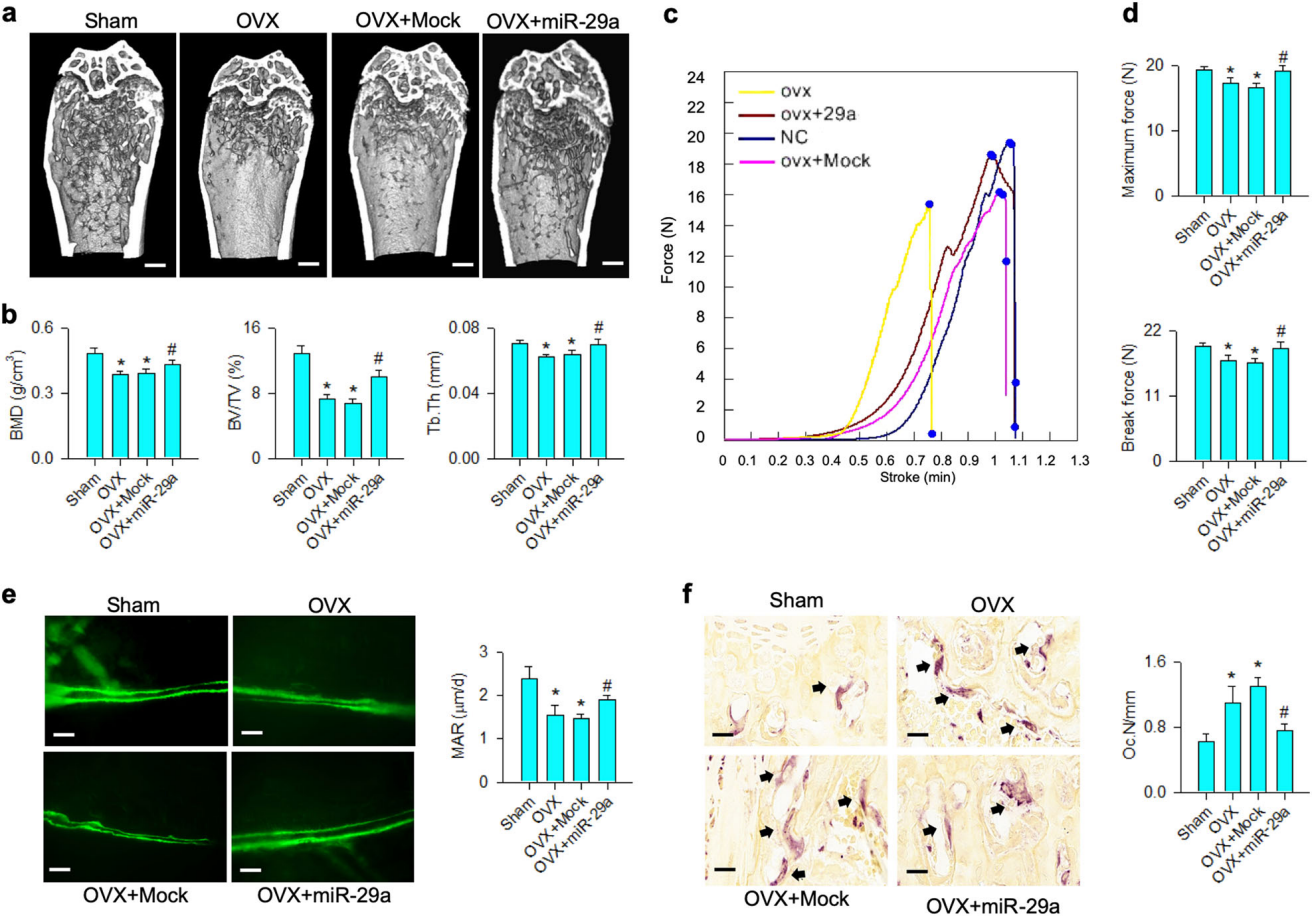
miR-29a precursor treatment for bone microstructure and histology. miR-29a precursor attenuated the ovariectomy-induced loss of trabecular microarchitecture (**a**); scale bar: 5 mm; BMD, BV/TV, and Tb.Th (**b**), as well as improved load-displacement profile (**c**), maximum force and breaking force (**d**). The treatment retained fluorescent calcein accumulation (scale bar: 40 μm) and MAR (**e**) and compromised the ovariectomy upregulation of osteoclast distribution (scale bar: 8 μm) and Oc.N (**f**). The data are expressed mean ± SEM calculated from eight mice. Asterisks * and hashtags # indicate significant difference from sham controls and OVX, respectively

Given that bone loss was improved in ovariectomized miR-29aTg/OCN mice, we further asked whether treatment with miR-29a precursor affected the estrogen deficiency-induced osteoporosis. To this end, lentivirus miR-29a precursor was injected into ovariectomized mice. It significantly compromised the severity of trabecular microarchitecture loss (Fig. 5a), reversed BMD, BV/TV, and Tb.Th (Fig. 5b) along with improved mechanical properties, including maximum force (Fig. 5c) and breaking force, (Fig. 5d) of skeletons in ovariectomized mice. Injection of miR-29a precursor also retained mineral accumulation (Fig. 5e), but downregulated the ovariectomy-mediated excessive osteoclast-formation histopathology (Fig. 5f).

### miR-29a inhibited expression of pro-osteoclast regulators in osteoblasts

**Fig. 6:**
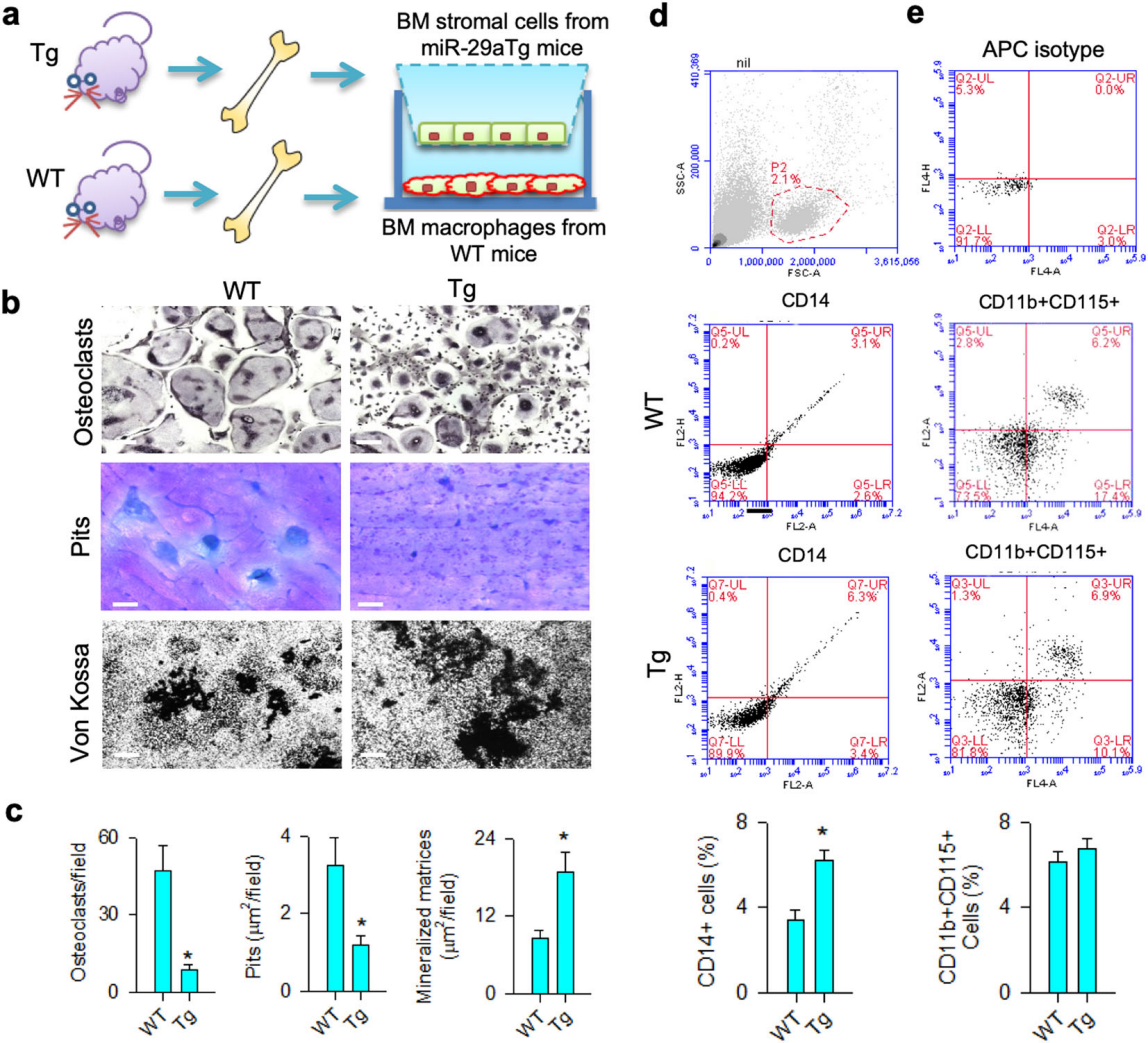
Osteoclast-formation capacity of bone marrow macrophages in miR-29aTg/OCN mice. Schematic drawing of bone marrow macrophages co-incubated with bone marrow mesenchymal stem cells (**a**). Decreased osteoclast differentiation (scale bar: 8 μm) and pit formation (scale bar: 20 μm) of wild-type bone marrow macrophage and increased mineralized matrix formation (scale bar: 40 μm) of wild-type bone marrow cells co-incubated with miR-29aTg/OCN bone marrow mesenchymal cells (**b, c**). Investigations are expressed mean ± SEM calculated from six mice. High CD14 + macrophages (**d**) rather than CD11 + CD115 + monocytes (**e**) existed in bone marrow in miR-29aTg/OCN mice. Investigations are expressed mean ± SEM calculated from four mice. Asterisks * indicate significant difference from wild-type mice

As a low osteoclast-formation reaction occurred in miR-29aTg/OCN mice, we wondered whether miR-29aTg/OCN bone marrow osteogenic cells affected these activities. Analysis of co-culture models (Fig. 6a) confirmed an increase in mineralized matrix formation of miR-29aTg/OCN bone marrow mesenchymal cells, which resulted in significant reductions in osteoclast differentiation and pit formation of WT bone marrow macrophages (Fig. 6b, c). These investigations prompted us to further quantify osteoclastogenic precursor cells in bone marrow in these mice. CD14-postive macrophages were significantly increased in miR-29aTg/OCN mice (Fig. 6d), whereas monocytes positive for CD11b and CD115 in bone marrow nucleated cells were comparable with WT mice (Fig. 6e), which is indicative of miR-29a may block osteoclastogenic differentiation reaction.

### miR-29a inhibited RANKL and CXCL12 expression in osteoblasts

**Fig. 7:**
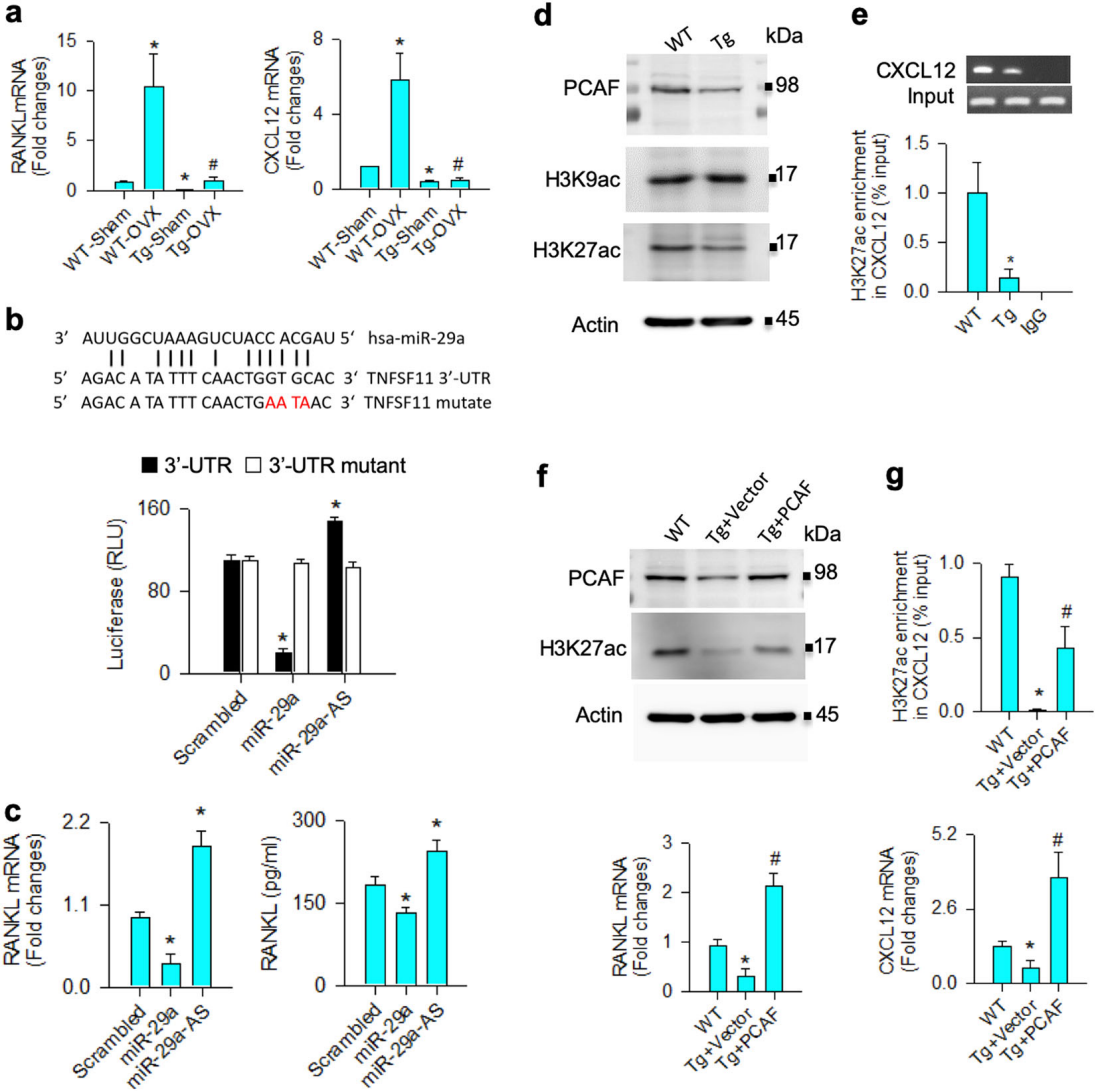
Analysis of miR-29a inhibition of RANKL and CXCL12 expression in bone marrow mesenchymal cells. miR-29a compromised the ovariectomy upregulation of RANKL and CXCL12 expression in bone marrow mesenchymal cells (**a**). The data are expressed mean ± SEM calculated from six mice. Asterisks * and hashtags # indicate significant difference from sham controls and OVX, respectively. Forced miR-29a expression decreased luciferase activity of 3′-UTR of RANKL mRNA (**b**) and downregulated RANKL mRNA expression and protein abundances, whereas miR-29 interference upregulated RANKL expression (**c**) of mesenchymal stem cell cultures. miR-29a overexpression reduced PCAF and H3K27ac abundances (**d**) and decreased H3K27ac enrichment in CXCL12 promoters (**e**). Forced PCAF expression repressed the miR-29a downregulation of H3K27ac levels and RANKL expression (**f**). It increased H3K27ac occupancy in CXCL12 promoters and CXCL12 expression (**g**) in bone marrow mesenchymal cells. Investigations are expressed mean ± SEM calculated from three experiments. Asterisks * and hashtags # indicate significant difference from WT and miR-29aTg/OCN, respectively

We investigated how miR-29a regulated osteoblasts to reduce osteoclast formation. RANKL and CXCL12 produced by osteogenic cells are important factors controlling osteoclast differentiation^5,7^. Significantly increased RANKL and CXCL12 expression of bone marrow mesenchymal cells occurred in ovariectomized WT mice. Of note, the expression of these two osteoclastogenic factors was compromised in ovariectomized miR-29aTg/OCN mice (Fig. 7a).

Bioinformatics shows that RANKL is a putative target of the miR-29 family (www.TargetScanHuman.org/vert_71/). In this study, forced miR-29a expression significantly decreased 3′-UTR luciferase activity (Fig. 7b), mRNA and protein expression of RANKL (Fig. 7c), whereas the luciferase reporter activity and RANKL expression were upregulated upon knocking down miR-29a in bone marrow mesenchymal stem cell cultures (Fig. 7b, c). miR-29a did not significantly change the luciferase activity of 3′-UTR mutate, which is suggestive of that this microRNA directly targeted RANKL mRNA.

miR-29a is shown to alter the acetylation status of histones in various cell types^19–21^. In bone marrow mesenchymal cells, the abundances of histone acetyltransferase PCAF and acetylated lysine 27 of histone 3 (H3K27ac) rather than H3K9ac levels were decreased in miR-29aTg/OCN mice (Fig. 7d). miR-29a overexpression also downregulated the H3K27ac enrichment in CXCL12 promoters as evident from chromatin immunoprecipitation–PCR assay (Fig. 7e). Forced PCAF expression reversed the miR-29a-induced H3K27ac loss, but increased RANKL expression (Fig. 7f). It also increased the H3K27ac occupancy in CXCL12 promoters, as well as increased CXCL12 along with augmented RANKL expression in bone marrow mesenchymal cells (Fig. 7g).

## Discussion

Excessive bone turnover is a notable feature of osteoporotic skeletons as resorption activity is far more than osteoblastic bone mineral gain in skeletal tissue^22,23^. A dysregulated interaction between bone-forming cells and skeleton-resorbing cells is known to impede bone mass homeostasis^24^. For example, osteoblasts and osteocytes in osteoporotic bone in aged or ovariectomized mice overproduce FasL^25^, connexin-43 and osteoclastogenic factors^26^, which enhance osteoclast differentiation and activation. Osteoclasts secrete extracellular matrices^27^ and exosomal microRNA adverse to osteoblast differentiation and bone formation^28^. The epigenetic pathways underlying the osteoblast regulation of osteoclast behavior during osteoporotic skeleton development is poorly understood. This study is the first indication that reveals the inhibitory action of miR-29a in osteogenic cells to excessive bone remodeling and osteoporosis development. It repressed the H3K27ac-mediated RANKL and CXCL12 expression, resulting in a low osteoclastic activity. Investigations also highlight the perspective of miR-29a precursor treatment protective against osteoporotic diseases.

In this study, miR-29a is indispensable in osteoblast differentiation because knocking down this molecule decreased osteogenic maker expression and mineralized matrix formation below the baseline. On the other hand, a low miR-29a expression occurred in mice with estrogen deficiency-mediated bone loss, which also hinted the relevance of this microRNA to skeletal integrity. Analyses were in agreement with other groups showing that the miR-29 family is advantageous to osteoblast function and mineralized matrix synthesis^15,18,29^. The profound phenotypes of high bone mass, trabecular microstructure, and mineral acquisition rate in osteoblast-specific miR-29aTg/OCN mice also consolidated the manifestation that this microRNA has positive effects on bone formation and skeletal development.

However, still little known is what miR-29a signaling in osteoblasts may contribute to osteoclastogenic differentiation capacity. It was noteworthy that osteoclast differentiation and eroded pit formation were downregulated as bone marrow macrophages were co-incubated with miR-29a-transferred osteogenic cells, which was suggestive of an inhibitory action to osteoclasts. The function of miR-29 to osteoclast behavior appears to depend on the incubation conditions. For example, other groups report that knocking down miR-29 expression in bone marrow macrophages and murine RAW264.1 monocytic cells decreases osteoclast differentiation and actin ring formation^30^. Analysis of this study uncovered high osteoclast lineage specification of bone marrow macrophages upon co-incubating with osteogenic cells lacking miR-29a. Low osteoclast surface histology and decreased ex vivo osteoclast formation of bone marrow macrophages in miR-29aTg/OCN mice further underpinned the outlook for the miR-29a repression of osteoclasts. Investigations also indicated the complex nature of bone cell behavior in skeletal microenvironment.

Striking findings were that a plethora of estrogen deficiency-induced osteoporosis signs, like bone mass loss, porous trabecular microstructure and weak biomechanical properties, were effectively compromised in osteoblast-specific miR-29Tg/OCN mice, which is suggestive of an osteoporosis-inhibitory effect occurred in miR-29Tg/OCN mice. Consistent with the investigations of ovariectomized miR-29aTg/OCN mice, treatment with miR-29a precursor also enabled ovariectomized mice to retain bone mineral density and mechanical strength, delaying the progression of osteoporosis. In addition, miR-29aTg/OCN osteogenic cells inhibited WT macrophage precursor cells differentiating into osteoclasts, which was indicative of that miR-29a shielded skeletal tissue from excessive bone turnover. The miR-29a improvement of bone formation and resorption reactions prompted us to understand how miR-29a signaling modulated the interaction between osteoblasts and osteoclasts within the skeletal microenvironment.

Bone marrow macrophages^31^ along with osteoclastogenic factors^32^ contribute to osteoclast differentiation and bone resorption in various physiological and pathological statuses. In this study, an intricate nature of osteoclast behavior in miR-29aTg/OCN mice was that these animals had increased bone marrow CD14 + macrophages, which elicited a low osteoclast-formation capacity. Thus, we hypothesized that deregulated osteoclastogenic factor production may lead to a low osteoclast differentiation reaction in miR-29aTg/OCN mice. RANKL, OPG^33^, CXCL9^34^, and CXCL12^35^ secreted by osteogenic cells are shown to promote macrophages progressing toward osteoclastic cells. Of interest, RANKL and CXCL12 in bone marrow mesenchymal cells were decreased in miR-29aTg/OCN mice, which was suggestive of that miR-29a suppressed osteoclastogenic factor production, hindering macrophage differentiation into osteoclastic cells in skeletal environment. Analysis conveyed a new insight into molecular events by which miR-29a repressed osteoclast formation.

This study shed light on two different intracellular pathways participating in the miR-29a regulation of RANKL and CXCL12 expression in osteoblasts. A canonical pathway was that miR-29a targeted the 3′-UTR region of RANKL and resulted in RANKL underproduction. On the other hand, the acetylation state of histone assembly regulated by histone acetyltransferase and deacetylase is known to alter CXCL12 transcription in osteoblasts and synovial fibroblasts^36–38^. PCAF signaling is found to regulate the acetylation status of CXCL12 promoter^39^ and osteogenic differentiation of mesenchymal stem cell cultures^40^. Our study further uncovered a noncanonical pathway that miR-29a inhibition of PCAF interfered with CXCL12 expression through H3K27 hypoacetylation and thus downregulated the H3K27ac occupancy in CXCL12 promoter in osteoblast cultures. We do not rule out the possibility that miR-29a overexpression may change other signaling transduction, which decreases osteoclastogenic factor expression in osteoblasts or RANKL expression in osteocytes. The miR-29a inhibition of multiple osteoclastogenic factors in osteoblasts underscores its beneficial action to skeletal tissue integrity.

**Fig. 8:**
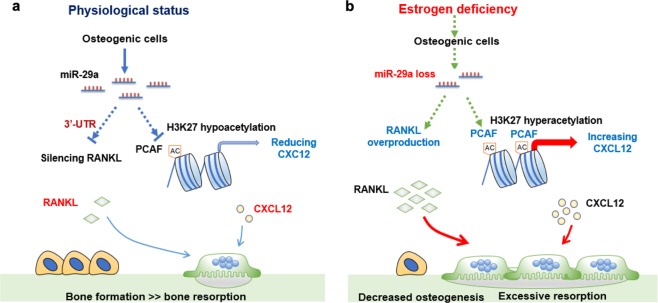
Schematic drawing of miR-29a signaling in osteoblasts repressed osteoclastic activity and osteoporosis. miR-29a targets RANKL expression and downregulates PCAF signaling-mediated CXCL12 expression to reduced osteoclastic activity, sustaining bone mass (**a**). Estrogen loss decreases miR-29a loss, which augments RANKL and CXCL12 expression in osteogenic cells, accelerating osteoclast formation and osteoporosis development (**b**)

Taken together, miR-29a signaling in osteoblasts is involved in orchestrating bone acquisition and erosion. It enhances osteogenic differentiation and bone formation, as well as targets RANKL and represses the H3K27ac-mediated CXCL12 expression, sustaining bone mass homeostasis (Fig. 8a). Estrogen deficiency induces miR-29a loss, which causes RANKL and CXCL12 overproduction to exaggerate osteoclastic resorption and ultimately provokes osteoporosis (Fig. 8b). This study offers productive insight into the biological roles of miR-29a in the interplay between osteoblasts and osteoclasts and reveals the remedial potential of miR-29a for improving osteoporotic disorders.

## Materials and methods

### Cell cultures

Animal use protocols were followed the animal well-being guidelines and approved by IACUC of Kaohsiung Chang Gung Memorial Hospital (Affidavit No. 2014120401). Eight-week-old male C57L/B6 mice were euthanatized, femurs and tibiae were dissected. Primary bone marrow mesenchymal cells and macrophage precursor cells were, respectively, isolated and incubated in basal medium containing DMEM with 10% fetal bovine serum as previously described^18^. In some experiments, GIBCO^®^-C57BL/6 immortalized murine bone marrow mesenchymal stem cells obtained from Thermo Fisher Scientific Inc. were maintained in the basal medium.

### In vitro osteogenic differentiation

Primary bone marrow mesenchymal cells (10^5^ cells/well) were grown in an osteogenic condition. Briefly, cell cultures were seeded in 12-well plates containing the basal medium, 50 μg/ml ascorbic acid and 1 mM β-glycerophosphate, and incubated in a humidified incubator at 37 °C, 5% O_2_ for 18 days. The osteogenic medium in each well was changed every 3 days. Upon incubation, mineralized matrix in cell cultures was probed using von Kossa-staining protocol. Three fields in each well and three wells in each specimen were selected to measure the matrix area (μm^2^/field) using microscopy along with an image analysis software (Zeiss)^41^.

### In vitro osteoclast and pit formation

Primary bone marrow macrophages were isolated, as previously described^41^. In brief, bone marrow nucleated cells were incubated in α-MEM, 10% FBS, and 20 ng/ml M-CSF for 1 day and then collected the floating cells. The non-adherent cells (2 × 10^4^ cells/well) were further incubated in a basal medium with 40 ng/ml RANKL and 20 ng/ml M-CSF (R&D Systems) for 10 days. Osteoclasts were detected using tartrate-resistant acid phosphatase staining. For pit-formation assay, macrophages were seeded onto bovine cortical bone slices (Boneslices.com) and incubated in the osteoclastogenic medium. After incubation, bone slices were rinsed with 1 M ammonium chloride and followed by 0.5% toluidine blue staining, as previously described^42^. Osteoclast number and pit area in three random fields of each specimen were quantified.

### Transwell co-culture

Transwell culture kits (Corning^®^ HTS 24-well plates, 0.4 μm, Thomas Scientific) were employed. In total, 2 × 10^4^/well bone marrow macrophages were seeded onto the lower wells containing the osteoclastogenic medium, 2 × 10^4^ bone marrow mesenchymal cells were incubated in the upper wells containing the osteogenic medium for 10 days. After incubation, osteoclast formation in the lower wells were subjected to tartrate-resistant acid phosphatase staining.

### In vitro transfection

In total, 1 × 10^6^ bone marrow mesenchymal stem cells (12-well plates; GIBCO®-C57BL/6 immortalized) were incubated in the basal medium and transferred with 25 nM miR-29a precursor, antisense oligonucleotide, or scrambled control (Applied Biosystems Inc, Foster City, CA) using Lipofectamine^TM^ 2000 (Invitrogen), according to the manufacturer’s manuals. The transfected cell cultures were incubated in the osteogenic medium for 18 days. In some experiments, cell cultures were transfected with expression vectors coding PCAF or empty vectors.

### Osteoblast-specific miR-29aTg/OCN mice

C57L/B6 mice overexpressing human miR-29a precursor transcribed by the osteoblast marker osteocalcin promoter were bred^15^. Their genotype expressing a 506-bp construct in genomic DNA isolated from tail tips was confirmed using primers (forward, 5′-GAGGATCCCCTCAAGGATACCAAGGGATGAAT-3′ and reverse, 5′-CTTCTAGAAGGAGTGTTTCTAGGTATCCGTCA-3′). Wild-type mice were littermates that did not express the construct of interest.

### Ovariectomized-induced osteoporosis

Twelve-week-old female miR-29aTg/OCN mice (*n* = 8) and wild-type mice (*n* = 8) were anesthetized, bilateral ovariectomy was performed with an aseptic surgery. For sham controls, miR-29aTg/OCN mice (*n* = 8) and wild-type mice (*n* = 8) received surgery without removing ovaries. Each animal was intraperitoneally given 25 μg/ml calcein to label mineral acquisition status at 3 and 9 days before euthanasia. At 4 weeks postoperatively, animals were euthanatized, tibiae and femurs were dissected for μCT scanning, biomechanics, and histological examination.

### In vivo lentivirus miR-29 precursor treatment

Lentivirus particles for pMIF-cGFP-zeo expressing miR-29a precursor were prepared and titrated to 1 × 10^7^/μl infectious units, as previously described^43^. At 1 week postoperatively, 100 μl lentivirus particle suspensions or vehicle were injected into ovariectomized mice via tail veins. Eighteen ovariectomized mice were divided into three groups to receive miR-29a precursor (*n* = 6), mock (*n* = 6), and vehicle (*n* = 6). Mice received sham operation were designated as sham controls (*n* = 6). At 4 weeks after ovariectomy, animals were euthanatized, bone tissue was dissected for studies.

### μCT analysis

The radiographic image (9-μm voxel size) acquisition and reconstruction of microstructure of proximal tibiae and L1–L5 lumbar vertebrae were performed with the μCT system (SkyScan 1176, Bruker) along with SKYSCAN® CT-Analysis software. The trabecular and cortical morphometry of the region of interest (300 slices of images), including BMD (g/cm^3^), BV/TV (%), B.Ar/T.Ar (%), Tb.Th (mm), Tb.N, connectivity (mm^2^), Tb.Sp (mm), SMI, and Ct.Th (mm), were measured according to the manufacturer’s manuals.

### Biomechanical property assay

Three-point bending assessment of femurs were performed using SHIMADZU EZ-SX Material Test System (Shimadzu Corporation) as a 50-N load was displaced (10 mm/min) and loaded to the specimens, which were hold onto 6-mm jag span holders. Displacement profile, breaking force (N), and maximum force (N) of specimens were computed using TRAPEZIVMX software.

### Histomorphometry

Fluorescent calcein accumulation and trabecular bone histology in methyl acrylate-embedded tibiae were probed using fluorescence microscopy and von Kossa staining, respectively. In some experiments, osteoclasts and osteoblasts in sections of decalcified specimens were probed using tartrate-resistant acid phosphatase and alkaline phosphatase staining, respectively. Mineral acquisition rate (μm/day), trabecular bone histology (BV/TV, %), osteoclast number (Oc.N/mm), and osteoblast number (Ob.N/mm) were measured using Zeiss image analysis software. Three random fields in each section, three sections of each mouse, and six animals were randomly selected for analysis.

### RT-quantitative PCR

Trizol regent and ReadScript^®^ Two-Step cDNA Synthesis Kits (Sigma-Aldrich) were utilized to extract the total RNA from 10^6^ cell cultures and reversely transcribe 1 μg total RNA, respectively. mRNA of interest was probed using the ABI 7900 Detection System (Applied Biosystems), 2 × TaqMan^®^ Universal PCR Master Mix along with primers for Runx2, osteocalcin, RANKL, CXCL12, and actin (Supplementary Table 1). The threshold value (Ct) of each gene was computed automatically. Fold change of mRNA expression was calculated according to the equation, 2^-ΔΔCt^, as previously described^42^. In some experiments, miR-29a and U6 expression in specimens was probed using primers (Ambion), SYBR Green FastMix reagent, and ABI 7900 Detection System (Applied Biosystems).

### Flow cytometry

Bone marrow in tibiae and femurs were aspirated from miR-29aTg/OCN mice and wild-type mice after euthanasia, mixed with ammonium–chloride–potassium buffer to isolate nucleated cells. Macrophages in 10^6^ bone marrow nucleated cells were probed with CD14 antibody conjugated with PE along with CytoFix/CytoPerm Kits (BD Biosciences) and quantified using flow cytometry (BD Accuri C6, San Jose, CA, USA). In a subset experiment, monocytes were probed with CD11 antibody conjugated with PE and CD115 antibody conjugated with allophycocyanin. Cells probed with IgE-conjugated or allophycocyanin were designated as isotype controls.

### Luciferase reporter assay

Luciferase reporter pCRII-TOPO II (Invitrogen) containing wild-type (5′-AUUGGCUAAAGU CUACCACGAU-3′; NM_001038495.1) and 4-base pair mutant (5′-AGACATATT TCAACTG*AATA*AC-3′) 3′-UTR of RANKL were constructed, respectively. In total, 10^4^ bone marrow mesenchymal stem cells in each well (96-well plates) were transfected with 10 ng luciferase reporter along with 10 ng Renilla luciferase reporter (Invitrogen). In all, 30 nM miR-29a precursor, antisense oligonucleotide and scrambled control were further transfected into the cell cultures. Detection of luciferase activity normalized with Renilla luciferase reaction was performed using Dual Luciferase Detection Kits (Promega) along with fluorescence spectrophotometer.

### Western blotting

PCAF, H3K9ac, H3K27ac, and actin abundances in cell lysates isolated from 5 × 10^6^ cells were detected with immunoblotting protocols probed by respective primary antibodies (Cell Signaling Technology, Danvers, MA, USA). Proteins of interest on the blots were visualized using IgG-conjugated horseradish peroxidase along with LumiGLO® chemiluminescent agent and H_2_O_2_ (Cell Signaling Technology).

### Chromatin immunoprecipitation (ChIP)-PCR

H3H27ac immunoprecipitates in 10^7^ cells were isolated using H3K27ac antibody and IgG together with Megna ChIP™ A/G Chromatin Immunoprecipitation (ChIP) kits (Millipore, Temecula, CA, USA). The immunocomplexes were further processed with proteinase K and sonication to extract DNA. In all, 1 ng DNA specimen was subjected to PCR analysis using Cy3-conjuated probes (Supplementary Table 1) for the -1991~+ 8 bp region proximal to CXCL12 promoter (ENSMUSG00000061353), respectively. PCR assay of GADPH promoter (ENSMUSG00000207654) was designated as positive controls. H3K27ac enrichment to the promoter of interest was expressed as % input DNA.

### Statistical analysis

Investigations among sham wild-type mice, ovariectomized wild-type mice, sham miR-29aTg/OCN mice, and ovariectomized miR-29aTg/OCN mice were analyzed using an ANOVA test and followed by a Bonferroni post hoc test. Statistical difference was reached as *P*-value < 0.05.

## Supplementary information

Supplementary Table 1
