# Supplementary material for: MicroRNA-29a represses osteoclast formation and protects against osteoporosis by regulating PCAF-mediated RANKL and CXCL12
Source: Cell Death Dis. 2019 Sep 23;10(10):705. doi: 10.1038/s41419-019-1942-1 (PMC6755134; doi:10.1038/s41419-019-1942-1)
Supplement: Supplementary file 1 — Supplementary Table 1 [file 41419_2019_1942_MOESM1_ESM.docx]

**Supplementary Table 1**

**Oligonucleotide sequences for RT-quantitative PCR and ChIP-PCR assays**

| **Oligonucleotide sequences for RT-qPCR primers** | |
| --- | --- |
| miR-29a | UCACAGAACCGGUCUCUUU |
| U6 | GTGCTCGCTTCGGCAGCACATATACTAAAATTGGAACGATACAGAGAAGATTAGCATGGCCCCTGCGCAAGGATGACACGCAAATTCGTGAAGCGTTCCATATTTT |
| Runx2 | Forward: 5’-CCAGCAGCACTCCATATCTC-3’  Reverse: 5’-CAGCGTCAA CACCATCATTC -3’ |
| Osteocalcin | Forward: 5’-CAAGCAGGGAGGCAATAAGG-3’  Reverse: 5’-CGTCAC AAGCAGGGTTAAGC-3’ |
| RANKL | Forward: 5’-CATCGGGTTCCCATAAAG-3’  Reverse: 5’- AAAGCAAATGTTGGCGTA-3’ |
| CXCL12 | Forward, 5’-CTTCATCCCCATTCTCCTCA-3’;  Reverse: 5’-GACTCTGCTCTGGTGGAAGG-3’ |
| Actin | Forward: 5’-GACGGCCAGGTCATCACTAT-3’  Reverse: 5’-CTTCTGCAT CCTGTCAGCA A-3’ |
| **Oligonucleotide sequences for ChIP-PCR primers** | |
| CXCL12 | Forward: 5’-TGCCTAGGAGCATCTGGTCT-3’  Reverse: 5’-TCCATTTCTACCGGCTTTTG -3’ |
| GADPH | Forward: 5’-TACTAGCGGTTTTACGGGCG-3’  Reverse: 5’-TCGAACAGGAGGAGCAGAGAGCGA-3’ |
